# Plasma vitamin D and risk of colorectal cancer: the Japan Public Health Center-Based Prospective Study

**DOI:** 10.1038/sj.bjc.6603892

**Published:** 2007-07-10

**Authors:** T Otani, M Iwasaki, S Sasazuki, M Inoue, S Tsugane

**Affiliations:** 1 Epidemiology and Prevention Division, Research Center for Cancer Prevention and Screening, National Cancer Center, 5-1-1 Tsukiji, Chuo-ku, Tokyo 104-0045, Japan

**Keywords:** plasma 25-hydroxyvitamin D, colorectal cancer, nested case–control study

## Abstract

We investigated the association between plasma 25(OH)D and the subsequent colorectal cancer incidence risk by a nested case–control study in The Japan Public Health Center-based Prospective Study, covering 375 newly diagnosed cases of colorectal cancer from 38 373 study subjects during a 11.5-year follow-up after blood collection. Two controls were matched per case on sex, age, study area, date of blood draw, and fasting time. In a conditional logistic regression model with matched pairs adjusted for smoking, alcohol consumption, body mass index, physical exercise, vitamin supplement use, and family history of colorectal cancer, plasma 25(OH)D was not significantly associated with colorectal cancer in men or in women. However, the lowest category of plasma 25(OH)D was associated with an elevated risk of rectal cancer in both men (odds ratio (OR), 4.6; 95% confidence interval (CI), 1.0–20) and women (OR, 2.7, 95% CI, 0.94–7.6), compared with the combined category of the other quartiles. Our results suggest that a low level of plasma 25(OH)D may increase the risk of rectal cancer.

Ecologic studies have reported that sunlight or solar ultraviolet B exposure is inversely associated with the risk of colorectal cancer incidence and mortality in the United States (Grant and Garland, 2004; Giovannucci, 2005) and in Japan (Mizoue, 2004). This ultraviolet B is involved in the production of vitamin D from 7-dehydrocholesterol in the skin (Holick, 2004). Vitamin D, which is derived from skin and dietary products or supplemental sources, is catalysed to 25-hydroxyvitamin D in the liver, which is the most useful measure of vitamin D status (Hunter, 1998). The 25-hydroxyvitamin D re-enters the circulation and is converted in the kidney by 25-hydroxyvitamin D-1*α*-hydroxylate to 1,25-dihydroxyvitamin D, which regulates calcium metabolism through its interaction with its major target tissues, bone and intestine; 25-hydroxyvitamin D is also metabolised in colorectal mucosa for regulation of cellular growth.

Several prospective studies reported that 25-hydroxyvitamin D in the blood was inversely associated with colorectal cancer risk (Garland *et al*, 1989; Braun *et al*, 1995; Wactawski-Wende *et al*, 2006), and especially distal colon and rectal cancer (Tangrea *et al*, 1997; Feskanich *et al*, 2004). One cohort was used to analyse a small number of cases (Garland *et al*, 1989; Braun *et al*, 1995), while others were from specific populations such as Finnish male smokers (Tangrea *et al*, 1997) and US nurses (Feskanich *et al*, 2004). Further confirmation is needed from general populations, especially with a different sunlight exposure and skin pigmentation reducing the cutaneous synthesis of vitamin D (Clemens *et al*, 1982; Matsuoka *et al*, 1991).

We investigated the association between plasma 25-hydroxyvitamin D and the subsequent risk of colorectal cancer in a nested case–control study in a large general population cohort in Japan.

## MATERIALS AND METHODS

The Japan Public Health Centre-based Prospective Study (JPHC study) is an ongoing cohort study investigating cancer, cardiovascular disease, and other lifestyle-related diseases. The first group (Cohort I) of the JPHC study started in 1990 and the second group (Cohort II) in 1993 (Watanabe *et al*, 2001). Study subjects were mainly residents living in several municipalities in each area administered by a Public Health Center, aged 40–59 years for Cohort I and 40–69 years for Cohort II. Two more subcohorts of health check-up examinees and random samples from one city aged 40–69 were added to Cohort II. The study subjects were identified by the population registry in each municipality. We studied a cohort of 65 803 men and 67 520 women. Our study was approved by the institutional review board of the National Cancer Centre, Tokyo, Japan.

Using a self-administered questionnaire, study subjects were asked to provide information about their personal and familial medical histories, smoking, alcohol consumption, frequency of physical exercise, dietary habits, and other lifestyle factors. Their dietary habits were assessed by a food-frequency questionnaire of 44 items for Cohort I (Tsubono *et al*, 2003) and 52 items for Cohort II. A total of 50 456 men (77%) and 55 909 women (83%) filled out and returned the questionnaire. Among the study subjects, 15 258 men (23%) and 26 703 women (40%) donated 10-ml of venous blood, drawn into vacutainer tubes containing heparin, collected at the time of their health check-ups (1990–1992 for Cohort I, and 1993–1995 for Cohort II), and divided into plasma and buffy layers, and then preserved at −80°C until analysis.

### Follow-up

We followed study subjects until 31 December, 2003, obtaining mortality details from the Ministry of Health, Labour and Welfare as necessary. Subjects moving to other municipalities were identified annually through residential registries in their Public Health Center areas; 9.9% moved away, and 0.2% were lost to follow-up.

Incidence data on colorectal cancer were collected for the JPHC cancer registry through local major hospitals, and population-based cancer registries. Indicators of the completeness of colorectal cancer case ascertainment conformed to the international standard (Parkin *et al*, 2002) as follows: 5.5% of incident cases were notified by death certificates (Death Certificate Notification, DCN); 2.2% did not have detailed information except death certificates (Death Certificate Only, DCO); and 94.7% were verified by histological examination (Histological Verification, HV). We identified 375 cases (196 men and 179 women) of colorectal cancer up to 31 December, 2003 from among the 38 373 subjects (14 004 men and 24 369 women) who had returned the baseline questionnaire, did not report diagnosis of any cancer, and provided the blood samples. All 375 cases were pathologically confirmed as adenocarcinoma, after excluding 18 cases of unknown pathology and seven non-adenocarcinoma cases. Of these, 256 subjects had cancer of the colon (International Classification of Diseases for Oncology, Third edition (ICD-O-3) (World Health Organisation, 2000) code C180–C189) and 119 had cancer of the rectum (ICD-O-3 code C199 and C209). Colon cancers were classified into proximal (ICD-O-3 code C180–C185) or distal colon (ICD-O-3 code C186 and C187). Information on tumour depth was available in 370 of the 375 cases, with 120 tumors of the intramucosal type corresponding to Tis in TNM classification (International Union Against Cancer, 1997) and 250 of the invasive type corresponding to T1 or more.

For each case, two controls were selected, using incidence density sampling (Clayton and Hills, 1993), matched on sex, age (within 3 years), date of blood draw (within 3 months), time since last meal (within 4 h), and study location (each Public Health Centre area) from subjects who had no history of colorectal cancer when the case was diagnosed.

Plasma 25-hydroxyvitamin D concentrations were measured by the competitive protein-binding assay of Haddad and Chyu (1971). A modified method using Gc-globulin (Sigma-Aldrich Co., St Louis, MO, USA) instead of a tissue extract was adopted. All samples were assayed by one commercial laboratory (Mitsubishi Kagaku Bio-Clinical Laboratories Inc., Tokyo, Japan). Samples from matched sets were assayed together. All laboratory personnel were blinded with respect to case or control status. The intra-assay coefficient of variation from the quality control samples was 8.4% (*n*=9).

### Statistical analysis

Adjusted means for cases and controls were calculated using least square means in analysis of covariance by the PROC GLM procedure in SAS software (version 9.1; SAS Institute Inc., Cary, NC, USA). Percentages of baseline characteristics were unadjusted crude values. We used the extensions of the Mantel–Haenszel procedure (Mantel, 1963) with matched pairs for a comparison of the baseline characteristics and plasma 25-hydroxyvitamin D between cases and controls, using the PROC FREQ procedure with CMH option. We tested the linear trend of covariates among controls by quartiles of plasma 25-hydroxyvitamin D, also using the extensions of the Mantel–Haenszel procedure (Mantel, 1963). The odds ratios (OR) and 95% confidence intervals (CI) for plasma 25-hydroxyvitamin D divided into quartiles based on control distribution were calculated by a conditional logistic regression model adjusted for pack–years of smoking (continuous), alcohol consumption (continuous), body mass index (continuous), physical exercise (less than once a week, or once a week or more), vitamin supplement use (any vitamin supplements, i.e., B-vitamins, vitamin C, E, A, multivitamins, or other), and family history of colorectal cancer as well as using matched pairs. The linear trend of OR was tested using the logarithmic-transformed median value of plasma 25-hydroxyvitamin D in the category, since the plasma value was log-normally distributed. The heterogeneity over quartiles of plasma 25-hydroxyvitamin D levels was tested by the Wald *χ*^2^ statistic. *P*-values for the trend were two-sided, with 0.05 as the significance level. We estimated the ORs for colorectal cancer as a whole, and also colon and rectal cancers separately with assessment of difference between these two cancers using a test for heterogeneity (Greenland, 1998). To examine whether the plasma 25-hydroxyvitamin D levels affected the risk of colorectal cancer differently between periods of high (July to November) and low (December to June) blood levels, we conducted a seasonally stratified analysis of blood drawn. Additionally, we tried to estimate the risk for hypovitaminosis D (15 ng ml^−1^ (37.5 nmol l^−1^)) (Nesby-O'Dell *et al*, 2002).

## RESULTS

Colorectal cancer cases in men smoked more, consumed more alcohol beverages, and had a higher body mass index than their controls, but also consumed less dietary fiber than controls (Table 1). Female cases consumed more diet–origin vitamin D than their controls.

No potential confounding factors correlated with plasma 25-hydroxyvitamin D among controls (Table 2). Female controls in the lowest quartile of plasma 25-hydroxyvitamin D consumed more alcoholic beverages than other quartiles. Food and nutrient intakes were not associated with plasma 25-hydroxyvitamin D except *n*-3 polyunsaturated fatty acid intake in women, which is contained in fish, a major source of dietary vitamin D in the Japanese population (Nakamura *et al*, 2002). Dietary vitamin D did not correlate with plasma 25-hydroxyvitamin D in either men or women.

Plasma 25-hydroxyvitamin D was lower in rectal cancer cases than their controls (Table 3). Median plasma levels were 24.3 ng ml^−1^ in men and 26.6 ng ml^−1^ in their controls (*P=*0.0051); 20.6 ng ml^−1^ in women and 22.6 ng ml^−1^ in their controls (*P=*0.093). There was no difference between colon cancer cases and their controls.

Plasma 25-hydroxyvitamin D was not associated with the risk of colorectal cancer in men or women, although there was a suggestion of an inverse relationship in men (Table 4), with ORs (95% CI) of 0.76 (0.42–1.4) for the second, 0.76 (0.39–1.5) for the third, and 0.73 (0.35–1.5) for the highest quartile, compared to the lowest quartile (*P* for trend, 0.39). It appeared that only the lowest quartile had a somewhat higher risk than other quartiles for rectal cancer risk, although again the trend test was not significant. The ORs were 1.0 for the lowest, 0.17 for the second, 0.25 for the third, and 0.075 for the highest quartile for men (*P* for trend, 0.06); colon cancer did not show a similar association. In women, a similar association was observed for rectal cancer risk, with ORs of 0.26, 0.46, and 0.33 for the second, third and highest quartiles respectively (*P* for trend, 0.17). Heterogeneity tests between colon and rectal cancer risk were borderline (*P*=0.06 in men; 0.04 in women), using estimates from the statistical model for the trend test.

In addition to the main results, we calculated the rectal cancer risk of the lowest quartile compared with the combined category of other quartiles in men and women. These ORs were 4.6 (95% CI, 1.0–20) in men and 2.7 (95% CI, 0.94–7.6) in women. The fact that the low plasma levels were associated with rectal cancer risk did not substantially change when the first 2-year cases were excluded, although the OR in men was attenuated (OR, 2.2 (95% CI, 0.44–11) in men; OR, 2.7 (95% CI, 0.92–7.8) in women). Further adjustment for *n*-3 polyunsaturated fatty acid or fish intake did not change the main results (data not shown).

We repeatedly examined the association after stratifying data by the season of blood collection, that is, that with high plasma levels (July to November) or low plasma levels (December to June), and by study location (northern or southern Japan), but the results did not substantially change. These further analyses could not be applied to rectal cancer separately because the numbers were too small; similarly the risk of hypovitaminosis D could not be investigated because of the small numbers (six men cases and six men controls; 10 women cases and 28 women controls).

## DISCUSSION

Our results suggest that a low level of plasma 25-hydroxyvitamin D is associated with rectal cancer risk in both men and women but not colon cancer. A significant inverse association between 25-hydroxyvitamin D and colorectal cancer, especially of the distal colon and rectum has been reported (Tangrea *et al*, 1997; Feskanich *et al*, 2004; Wactawski-Wende *et al*, 2006), whereas another prospective study showed no such association with colon cancer risk (Braun *et al*, 1995).

Comparison of the ranges of 25-hydroxyvitamin D levels in the above studies is relevant. A Finnish cohort (Tangrea *et al*, 1997) covered the lower and narrower range, that is 9.8 or less to more than 19.2 ng ml^−1^, as did the Women's Health Initiative Study, a randomised, double-blind, placebo-controlled trial among US women, from less than 12.4–23.4 ng ml^−1^ or more (Wactawski-Wende *et al*, 2006). A Nurses' Health Study among US women (Feskanich *et al*, 2004) covered a higher and wider range (14.9, the median for the lowest category to 35.3 ng ml^−1^, the median for the highest category). On the other hand, the small Washington County Study covered a higher and narrower range (less than 17.2–30.1 ng ml^−1^ or more) (Braun *et al*, 1995).

Our study similarly showed no association between colon cancer and 25-hydroxyvitamin D in the higher and narrower range, that is, below 22.9–32.1 ng ml^−1^ or more in men and below 18.7–27.0 ng ml^−1^ or more in women. However, an effect of a low level of 25-hydroxyvitamin D was found for rectal cancer in our subjects. In short, populations covering the lower range of 25-hydroxyvitamin D may show a preventive effect against colorectal cancer even with a narrower variation of 25-hydroxyvitamin D. The higher range presumably shows the protective effect only if they have a sufficiently wide variation of 25-hydroxyvitamin D. Differences in our findings from the Washington County Study may be due to the different characteristics of the colon and rectum.

Additionally, this inconsistency may reflect assay differences. Some studies used radioimmunoassay with an iodine-125 labelled tracer (Braun *et al*, 1995; Tangrea *et al*, 1997; Feskanich *et al*, 2004); others used a chemiluminescent radioimmunoassay (Wactawski-Wende *et al*, 2006) or competitive protein-binding assay (our study). If assay methodology differs among laboratories, even the same samples may show different measurements (Binkley *et al*, 2004).

Differences between colon and rectum may derive from 1,25-dihydroxyvitamin D receptor (Vitamin D receptor, VDR) expression. VDR also has some differences in genetic polymorphisms by ethnic group. *Bsm*I B and short poly A alleles, for example, are more prevalent in Caucasians than in Japanese (Tokita *et al*, 1996; Ingles *et al*, 1997), and may be protective against colorectal cancer (Slatter *et al*, 2001). Japanese may be more vulnerable to rectal cancer due to the low prevalence of this protective VDR genotype.

The Women's Health Initiative study reported that calcium plus vitamin D supplementation did not decrease the subsequent risk of colorectal cancer over a follow-up of 7 years (Wactawski-Wende *et al*, 2006). However, the supplementation might influence only the group with low plasma levels, colorectal cancer risk among the group with the lowest level of plasma 25-hydroxyvitamin D being slightly, though not significantly, decreased.

It is not surprising that bioavailable vitamin D status did not correlate with dietary vitamin D intake. Holick (2004) indicated that over 90% of the vitamin D requirement comes from casual exposure to sunlight. Of course, blood levels of 25-hydroxyvitamin D partly reflect dietary intake of vitamin D (Nakamura *et al*, 2000, 2002), although our results did not show a positive correlation between dietary and plasma vitamin D levels, partly due to the low validity of the food frequency questionnaire. Spearman's correlation coefficients between dietary records and estimates from the food frequency questionnaire were 0.26 for men and 0.38 for women in Cohort I; 0.32 for men and 0.28 for women in Cohort II, assessed by volunteers from our cohorts (Ishihara *et al*, 2006). In addition, skin pigmentation is one of the determinants of 25-hydroxyvitamin D levels. Oriental people show lower cutaneous synthesis of vitamin D than white people (Matsuoka *et al*, 1991). In addition to sunlight, dietary intake, and skin pigmentation, season of blood sampling and subjects' body mass index may influence blood levels (Giovannucci, 2005; Giovannucci *et al*, 2006). Thus, 25-hydroxyvitamin D measurement is more appropriate way to assess entirely vitamin D status than dietary intake.

An active form of vitamin D, 1,25-dihydroxyvitamin D, was not investigated in the present study, as its half-life in the circulation (less than 4 h) is shorter than that of 25-hydroxyvitamin D (approximately 2 weeks) and normal levels are maintained even with vitamin D deficiency and low 25-hydroxyvitamin D levels, making it unsuitable for studies of colorectal cancer risk (Braun *et al*, 1995; Tangrea *et al*, 1997; Feskanich *et al*, 2004; Holick, 2004).

Although blood samples were collected before cancer diagnosis, measurement errors may exist because only one blood sample for each subject was used to measure plasma 25-hydroxyvitamin D concentrations, causing random misclassification to attenuate statistical associations. Long-term storage is recommended only at temperatures of less than −18°C (Hunter, 1998) and our plasma storage (−80°C) satisfied this condition. Furthermore, this plasma biomarker has a seasonal variation, declining in winter and rising in summer, reflecting sunlight exposure (Holick, 2004). In fact, our study controls' levels were slightly lower among blood samples drawn in winter (mean values from November to March; 26.3 ng dl^−1^ for men and 22.8 ng dl^−1^ for women) than those drawn at other times (mean values from April to October; 28.4 ng dl^−1^ for men and 23.5 ng dl^−1^ for women). To minimise bias, we matched date of blood collection between cases and controls (within 3 months), differing by less than 1 month among 63% of the case–control pairs, only by 1–2 months among 14% of them, and by 2–3 months among 17%. The proportion of case–control pairs where both were drawn at high-level seasons of plasma 25-hydroxyvitamin D (April to October) was 72% of all pairs, so difference in timing of blood draw would minimally impact on risk estimates.

In conclusion, a low plasma 25-hydroxyvitamin D level may be associated with a subsequent risk of rectal but not colon cancer.

## Figures and Tables

**Table 1:** Baseline characteristics of cases and controls

	**Men**			**Women**		
**Characteristics**	**Cases**	**Controls**	***P***	**Cases**	**Controls**	***P***
*n*	196	392		179	358	
Age (years), mean	56.9	56.9	0.69	56.5	56.4	0.35
Smoking (pack–years), mean^a^	27.5	23.8	0.020	0.458	0.657	0.54
Alcohol consumption (g week^−1^ ethanol), mean^a^	236	175	<0.0010	9.63	5.70	0.37
Body mass index (kg m^−2^), mean^a^	23.8	23.2	0.027	23.5	23.6	0.68
Physical exercise, *n* (%)^b^	49 (26)	75 (20)	0.12	33 (19)	54 (15)	0.28
Vitamin supplement use, *n* (%)	35 (20)	53 (15)	0.11	24 (15)	54 (17)	0.43
Family history of colorectal cancer, *n* (%)	5 (2.6)	4 (1.0)	0.16	4 (2.2)	4 (1.1)	0.32
Total energy intake (kcal day^−1^), mean^c^	2021	2064	0.34	1277	1265	0.67
Dietary fibre intake (g day^−1^), mean^d^	7.76	8.05	0.047	7.83	7.58	0.16
Folate intake (*μ*g day^−1^), mean^d^	328	329	0.60	298	291	0.31
Calcium intake (mg day^−1^), mean^d^	441	463	0.15	452	423	0.074
Vitamin D intake (*μ*g day^−1^), mean^d^	6.58	6.67	0.54	5.57	5.16	0.047
*n*-3 fatty-acid intake (*μ*g d^−1^), mean^d^	1.32	1.38	0.28	1.19	1.13	0.060
Red meat intake (g d^−1^), mean^d^	17.0	17.2	0.70	13.8	12.7	0.17
Fish intake (g d^−1^), mean^d^	62.2	62.0	0.88	46.7	43.6	0.15

a Adjusted for age.

b Number (percentage) of subjects doing physical exercise once a week or more.

c Adjusted for age and cohort.

d Adjusted for age, cohort, and energy intake.

**Table 2:** Association between plasma 25-hydroxyvitamin D and covariates among controls at baseline

	**Quartiles of plasma 25-hydroxyvitamin D**									
	**Men**					**Women**				
**Variables**	**Lowest**	**Second**	**Third**	**Highest**	***P* for trend**	**Lowest**	**Second**	**Third**	**Highest**	***P* for trend**
Range, ng ml^−1^	(<22.9)	(22.9–27.5)	(27.6–32.0)	(32.1+)		(<18.7)	(18.7–22.2)	(22.3–26.9)	(27.0+)	
Median, ng ml^−1^	19.9	25.0	29.6	35.6		16.6	20.6	24.1	31.4	
Age, mean	57.6	55.1	56.9	58.0	0.45	55.3	57.9	56.8	55.6	0.92
Smoking, mean^a^	25.9	23.9	21.4	23.9	0.44	0.8	1.3	0.4	0.2	0.22
Alcohol consumption, mean^a^	172	172	183	174	0.40	11.2	3.6	4.6	3.5	0.062
Body mass index, mean^a^	23.5	23.1	23.3	23.1	0.53	23.5	23.4	23.7	23.8	0.34
Physical exercise, *n*	18	21	16	20	0.70	11	16	12	15	0.83
Vitamin supplement use, *n*	15	9	21	8	0.30	14	15	12	13	0.74
Family history of colorectal cancer, *n*	0	1	1	2	0.15	0	2	1	1	0.47
Dietary fibre intake, mean^b^	7.53	8.56	8.39	7.64	0.84	7.13	7.60	7.62	7.80	0.96
Folate intake, mean^b^	338	340	331	312	0.091	279	288	292	300	0.48
Calcium intake, mean^b^	451	463	497	443	0.57	429	419	437	399	0.24
Vitamin D intake, mean^b^	6.30	6.84	7.14	6.47	0.92	5.00	5.19	4.84	5.54	0.067
*n*-3 fatty-acid intake, mean^b^	1.30	1.44	1.47	1.31	0.81	1.07	1.13	1.08	1.20	0.026
Red meat intake, mean^b^	17.8	17.8	16.2	17.0	0.50	10.6	13.8	13.4	12.9	0.49
Fish intake, mean^b^	59.5	63.9	66.2	58.9	0.70	42.2	44.0	39.1	48.4	0.087

a Adjusted for age.

b Adjusted for age, cohort, and energy intake.

**Table 3:** Plasma 25-hydvroxyvitamin D between cases and controls

	**Plasma 25-hydroxyvitamin D**, (**ng ml^−1^**)		
	**Cases**	**Controls**	***P* a**
*Men*			
Colorectal cancer, *n*	196	392	
Median [interquartile range]	27.3 [22.2–32.8]	27.6 [22.9–32.1]	0.67
Colon cancer, *n*	141	282	
Median [interquartile range]	28.3 [23.0–35.0]	28.0 [23.0–32.3]	0.25
Rectal cancer, *n*	55	110	
Median [interquartile range]	24.3 [20.2–29.1]	26.6 [22.9–31.2]	0.0051
			
*Women*			
Colorectal cancer, *n*	179	358	
Median [interquartile range]	22.5 [18.5–27.1]	22.3 [18.7–27.0]	0.91
Colon cancer, *n*	115	230	
Median [interquartile range]	23.3 [19.1–27.3]	22.0 [18.5–27.5]	0.25
Rectal cancer, *n*	64	128	
Median [interquartile range]	20.6 [17.8–25.3]	22.6 [19.1–26.5]	0.093

a Tested by extensions of Mantel–Haenszel procedure with matched pairs.

**Table 4:** Odds ratios (OR) and 95% confidence intervals (CI) of colorectal cancer for plasma 25-hydroxyvitamin D

	**Quartiles of plasma 25-hydroxyvitamin D**					
	**Lowest**	**Second**	**Third**	**Highest**	***P* for heterogeneity**	***P* for trend**
*Men*						
Range, ng ml^−1^	(<22.9)	(22.9–27.5)	(27.6–32.0)	(32.1+)		
Median, ng ml^−1^	19.9	25.0	29.6	35.6		
Colorectal cancer, *n*^a^	43/74	40/85	36/85	44/80		
Unadjusted OR^b^ (95% CI)	1.0^c^	0.81 (0.49–1.3)	0.64 (0.36–1.1)	0.91 (0.50–1.7)	0.37	0.53
Adjusted OR^d^ (95% CI)	1.0^c^	0.76 (0.42–1.4)	0.76 (0.39–1.5)	0.73 (0.35–1.5)	0.78	0.39
Colon cancer, *n*^a^	25/54	27/55	29/66	38/62		
Unadjusted OR^b^ (95% CI)	1.0^c^	1.0 (0.55–1.8)	0.89 (0.46–1.7)	1.5 (0.73–3.0)	0.41	0.37
Adjusted OR^d^ (95% CI)	1.0^c^	0.98 (0.48–2.0)	1.0 (0.48–2.3)	1.2 (0.51–2.7)	0.97	0.70
Rectal cancer, *n*^a^	18/20	13/30	7/19	6/18		
Unadjusted OR^b^ (95% CI)	1.0^c^	0.39 (0.15–1.0)	0.26 (0.084–0.82)	0.16 (0.036–0.69)	0.055	0.0066
Adjusted OR^d^ (95% CI)	1.0^c^	0.17 (0.024–1.2)	0.25 (0.051–1.3)	0.075 (0.0057–0.99)	0.19	0.06
						
*Women*						
Range, ng ml^−1^	(<18.7)	(18.7–22.2)	(22.3–26.9)	(27.0+)		
Median, ng ml^−1^	16.6	20.6	24.1	31.4		
Colorectal cancer, *n*^a^	41/77	34/73	44/71	41/76		
Unadjusted OR^b^ (95% CI)	1.0^c^	0.92 (0.54–1.6)	1.1 (0.61–1.8)	0.98 (0.51–1.9)	0.96	0.96
Adjusted OR^d^ (95% CI)	1.0^c^	1.0 (0.55–1.9)	1.2 (0.65–2.3)	1.1 (0.50–2.3)	0.92	0.74
Colon cancer, *n*^a^	21/53	27/48	27/41	31/53		
Unadjusted OR^b^ (95% CI)	1.0^c^	1.3 (0.68–2.5)	1.8 (0.88–3.6)	1.9 (0.83–4.3)	0.38	0.10
Adjusted OR^d^ (95% CI)	1.0^c^	1.7 (0.78–3.6)	2.1 (0.90–4.7)	2.1 (0.78–5.6)	0.34	0.12
Rectal cancer, *n*^a^	20/24	7/25	17/30	10/23		
Unadjusted OR^b^ (95% CI)	1.0^c^	0.39 (0.14–1.1)	0.39 (0.15–1.0)	0.28 (0.087–0.88)	0.14	0.04
Adjusted OR^d^ (95% CI)	1.0^c^	0.26 (0.069–1.0)	0.46 (0.15–1.4)	0.33 (0.084–1.3)	0.22	0.17

a Numbers of cases/controls.

b ORs estimated using matched pairs without any adjustment.

c Reference category.

d ORs estimated using matched pairs with adjustment for pack–years of smoking (continuous), alcohol consumption (g/week ethanol, continuous), body mass index (continuous), physical exercise (less than once a week, or once a week or more), vitamin supplement use, and family history of colorectal cancer.

## Appendix A

Members of the Japan Public Health Centre-based Prospective Study Group are: S Tsugane, M Inoue, T Sobue, T Hanaoka, National Cancer Centre, Tokyo; J Ogata, S Baba, T Mannami, A Okayama, National Cardiovascular Centre, Suita; K Miyakawa, F Saito, A Koizumi, Y Sano, I Hashimoto, Iwate Prefectural Ninohe Public Health Centre, Ninohe; Y Miyajima, N Suzuki, S Nagasawa, Y Furusugi, Akita Prefectural Yokote Public Health Centre, Yokote; H Sanada, Y Hatayama, F Kobayashi, H Uchino, Y Shirai, T Kondo, R Sasaki, Y Watanabe, Y Miyagawa, Nagano Prefectural Saku Public Health Centre, Saku; Y Kishimoto, E Takara, T Fukuyama, M Kinjo, M Irei, H Sakiyama, Okinawa Prefectural Chubu Public Health Centre, Okinawa; K Imoto, H Yazawa, T Seo, A Seiko, F Ito, F Shoji, Katsushika Public Health Centre, Tokyo; A Murata, K Minato, K Motegi, T Fujieda, Ibaraki Prefectural Mito Public Health Centre, Mito; K Matsui, T Abe, M Katagiri, M Suzuki, Niigata Prefectural Kashiwazaki and Nagaoka Public Health Centre, Kashiwazaki and Nagaoka; M Doi, A Terao, Y Ishikawa, Kochi Prefectural Chuo-higashi Public Health Centre, Tosayamada; H Sueta, H Doi, M Urata, N Okamoto, F Ide, Nagasaki Prefectural Kamigoto Public Health Centre, Arikawa; H Sakiyama, N Onga, H Takaesu, Okinawa Prefectural Miyako Public Health Centre, Hirara; F Horii, I Asano, H Yamaguchi, K Aoki, S Maruyama, M Ichii, Osaka Prefectural Suita Public Health Centre, Suita; S Matsushima, S Natsukawa, Saku General Hospital, Usuda; M Akabane, Tokyo University of Agriculture, Tokyo; M Konishi, K Okada, Ehime University, Matsuyama; H Iso, Y Honda, Tsukuba University, Tsukuba; H Sugimura, Hamamatsu University, Hamamatsu; Y Tsubono, Tohoku University, Sendai; M Kabuto, National Institute for Environmental Studies, Tsukuba; S Tominaga, Aichi Cancer Centre Research Institute, Nagoya; M Iida, W Ajiki, Osaka Medical Centre for Cancer and Cardiovascular Disease, Osaka; S Sato, Osaka Medical Centre for Health Science and Promotion, Osaka; N Yasuda, Kochi Medical School, Nankoku; S Kono, Kyushu University, Fukuoka; K Suzuki, Research Institute for Brain and Blood Vessels Akita, Akita; Y Takashima, Kyorin University, Mitaka; E Maruyama, Kobe University, Kobe; the late M Yamaguchi, Y Matsumura, S Sasaki, S Watanabe, National Institute of Health and Nutrition, Tokyo; and T Kadowaki, Tokyo University, Tokyo.
